# SMG1, a nonsense‐mediated mRNA decay (NMD) regulator, as a candidate therapeutic target in multiple myeloma

**DOI:** 10.1002/1878-0261.13343

**Published:** 2022-12-16

**Authors:** Alexander C. Leeksma, Ingrid A. M. Derks, Brett Garrick, Aldo Jongejan, Martino Colombo, Timon Bloedjes, Torsten Trowe, Jim C. Leisten, Michelle Howarth, Mehnaz Malek, Deborah S. Mortensen, Kate Blease, Mathew C. Groza, Rama Krishna Narla, Remco Loos, Marie‐José Kersten, Perry D. Moerland, Jeroen E. J. Guikema, Arnon P. Kater, Eric Eldering, Ellen H. Filvaroff

**Affiliations:** ^1^ Department of Hematology, Amsterdam University Medical Centers University of Amsterdam The Netherlands; ^2^ Department of Experimental Immunology Amsterdam University Medical Centers University of Amsterdam The Netherlands; ^3^ Lymphoma and myeloma center Amsterdam (LYMMCARE), Cancer Center Amsterdam (CCA) and Amsterdam Infection and Immunity Institute (AIII) The Netherlands; ^4^ Translational Research, Bristol Myers Squibb San Francisco CA USA; ^5^ Department of Clinical Epidemiology, Biostatistics and Bioinformatics Amsterdam University Medical Centers, University of Amsterdam The Netherlands; ^6^ Bristol Myers Squibb's Center for Innovation and Translational Research Europe (CITRE) Seville Spain; ^7^ Department of Pathology, Amsterdam University Medical Centers, Lymphoma and Myeloma Center Amsterdam (LYMMCARE) University of Amsterdam The Netherlands; ^8^ Discovery, Bristol Myers Squibb San Diego CA USA

**Keywords:** cancer, MM, NMD, SMG1, therapy, UPR

## Abstract

Early data suggested that CC‐115, a clinical molecule, already known to inhibit the mammalian target of rapamycin kinase (TORK) and DNA‐dependent protein kinase (DNA‐PK) may have additional targets beyond TORK and DNA‐PK. Therefore, we aimed to identify such target(s) and investigate a potential therapeutic applicability. Functional profiling of 141 cancer cell lines revealed inhibition of kinase suppressor of morphogenesis in genitalia 1 (SMG1), a key regulator of the RNA degradation mechanism nonsense‐mediated mRNA decay (NMD), as an additional target of CC‐115. CC‐115 treatment showed a dose‐dependent increase of SMG1‐mediated NMD transcripts. A subset of cell lines, including multiple myeloma (MM) cell lines sensitive to the endoplasmic reticulum stress‐inducing compound thapsigargin, were highly susceptible to SMG1 inhibition. CC‐115 caused the induction of UPR transcripts and cell death by mitochondrial apoptosis, requiring the presence of *BAX*/*BAK* and caspase activity. Superior antitumor activity of CC‐115 over TORK inhibitors in primary human MM cells and three xenograft mouse models appeared to be via inhibition of SMG1. Our data support further development of SMG1 inhibitors as possible therapeutics in MM.

## Graphical abstract

In this study, SMG1, a key regulator of the RNA degradation mechanism nonsense‐mediated mRNA decay (NMD), was discovered as an additional target of the clinical compound CC‐115. CC‐115 treatment showed a dose‐dependent increase of NMD transcripts, unfolded protein response (UPR) transcripts, and cell death via SMG1 inhibition in a subset of cell lines, primary multiple myeloma cells, and xenograft mouse models.

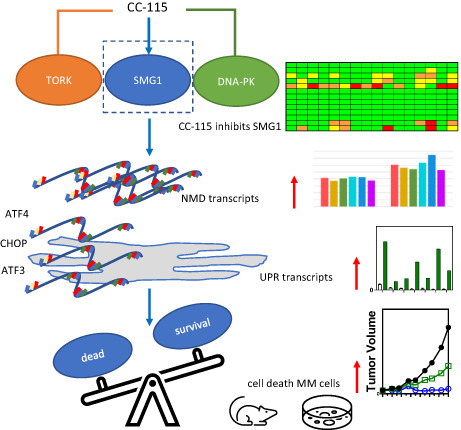

**Abbreviations**
AML: acute myeloid leukemia
ATCC: American Type Culture Collection
ATR: ataxia‐ and Rad3‐related
ATM: ataxia‐telangiectasia mutated
Bor: bortezomib
BL: Burkitt's lymphoma
CLL: chronic lymphocytic leukemia
DSMZ: Deutsche Sammlung von Mikroorganismen und Zellkulturen
DiOC6: Dihexyloxacarbocyanine Iodide
DMSO: dimethyl sulfoxide
DDR: DNA damage response
DNA‐PK: DNA‐dependent protein kinase
ER: endoplasmic reticulum
KO: knockout
LC–MS: liquid chromatography–mass spectrometry
MCL: mantle cell lymphoma
MM: multiple myeloma
NMD: nonsense‐mediated mRNA decay
PIKK: phosphoinositide 3‐kinase‐related kinase
PTCs: premature termination codons
PI: propidium iodide
QVD: quinoline‐val‐asp‐difluorophenoxymethylketone
S: serine
sgRNAs: single‐guide RNAs
SMG1i: SMG1 kinase inhibitor
SMG1: suppressor of morphogenesis in genitalia 1
TORK: target of rapamycin kinase
TG: thapsigargin
T: threonine
TRRAP: transformation/transcription domain‐associated protein
UPR: unfolded protein response
UPF1: UP‐frameshift protein 1

## 1 Introduction

Nonsense‐mediated mRNA decay (NMD) selectively degrades mRNAs containing premature termination codons (PTCs) and controls the expression of a subset of normal RNAs [1]. NMD is regulated by various types of cell stress, most of which are dysregulated in cancer [1, 2, 3, 4]. Alternative splicing is a potential source of PTC‐containing transcripts, which can give rise to aberrantly folded proteins, resulting in the activation of the unfolded protein response (UPR) [5, 6]. Initiation of UPR restores cellular function by halting protein translation, degrading misfolded proteins, and activating signaling pathways involved in the production of molecular chaperones for protein folding. Nonsense‐mediated mRNA decay and UPR have a complex and reciprocal interaction, which shifts in response to cell stress [7]. If the UPR is not resolved, then endoplasmic reticulum (ER) stress prevents recovery of cellular function, and the apoptotic pathway is initiated [8, 9].

Multiple myeloma (MM) is a heterogeneous and still fatal malignancy of plasma cells. Despite a variety of recent therapeutical developments, including immunomodulatory drugs, the development of resistance to proteasome inhibitors and other drugs in MM remains prevalent, and novel therapeutic targets and strategies are still warranted [10, 11].

CC‐115 was identified as an inhibitor of mammalian Target of Rapamycin Kinase (TORK) and DNA‐dependent protein kinase (DNA‐PK) [12, 13, 14]. In patients with solid tumors and hematologic malignancies, CC‐115 showed antitumor effects and was relatively safe, with toxicities similar to those of other TORK inhibitors [13, 15, 16]. TORK and DNA‐PK belong to the phosphoinositide 3‐kinase‐related kinase (PIKK) family, which includes ataxia‐telangiectasia mutated (ATM), ataxia‐ and Rad3‐related (ATR), suppressor of morphogenesis in genitalia (SMG1), and transformation/transcription domain‐associated protein (TRRAP) [17]. Generally, PIKK proteins have prominent roles in the DNA damage response (DDR) [18], while SMG1 is also a key NMD factor [19, 20]. SMG1 is critical for NMD as its kinase activity is required for the phosphorylation of UPF1 (UP‐frameshift protein 1). UPF1 is an essential component of a complex to recognize and initiate the degradation of PTC‐containing transcripts.

Following up on early data suggesting that CC‐115 may have additional targets beyond TORK and DNA‐PK, we characterized CC‐115 activity in a panel of cell lines and then profiled the binding activity of CC‐115 to > 200 kinases. In this way, we identified SMG1 as an additional target of CC‐115. The NMD process is highly complex and also variable, and not all mechanistic details are elucidated [2]. Nevertheless, inhibition of SMG1 kinase activity can be expected to stall or prevent NMD. In order to determine which tumor cells may be responsive to the SMG1 inhibitory effects of CC‐115, we tested various cancer cell types. MM cell lines were particularly sensitive to either CC‐115 or a more specific SMG1 inhibitor. SMG1 inhibition by CC‐115 decreased proliferation and survival in most MM cell lines, in primary MM cells, and in xenotransplant MM models. Mechanistic studies pointed to a UPR‐related mechanism for CC‐115‐based lethality via SMG1 inhibition, which is in line with MM cells being sensitive to ER‐stress‐induced UPR and cell death. Our data provide support for further clinical development of SMG1 as a therapeutic target in cancer.

## 2 Materials and methods

### 2.1 Primary cells and cell lines

This study was conducted in accordance with the Declaration of Helsinki and approved by the AMC Medical Committee on Human Experimentation. Primary MM cell samples were obtained upon informed consent from patients diagnosed at the Academic Medical Center, Amsterdam, The Netherlands. Cell lines were purchased from American Type Culture Collection (ATCC, Manassas, VA, USA), the Deutsche Sammlung von Mikroorganismen und Zellkulturen (DSMZ, Braunschweig, Germany), or Horizon Discovery Ltd [Cambridge, UK; HCT 116 Parental (#HD PAR‐082), and HCT 116 DNA‐PK^−/−^ (#HD R02‐049) cell lines]. Cells were cultured according to the vendor's recommendation.

### 2.2 Reagents

CC‐115, CC214‐1, and CC‐223 (TORK inhibitors) were synthesized in‐house (San Diego, CA USA). A previously described specific SMG1 kinase inhibitor [21], herein called SMG1i, was synthesized in‐house and also purchased from Bio‐Connect (Houston, TX, USA #QT213620). DNA‐PK inhibitor NU7441 was purchased from Selleckhem (Houston, TX, USA #S2638), bortezomib was obtained from Janssen‐Cilag (Tilburg, The Netherlands), and bleomycin (bleocin) was purchased from Merck (Cambridge, MA, USA #203408). Venetoclax (ABT‐199) was purchased from Active Biochem (Bonn, Germany). Thapsigargin was purchased from Sigma‐Aldrich Co. (St. Louis, MO, USA #T9033) and the pan‐caspase inhibitor Quinoline‐Val‐Asp‐Difluorophenoxymethylketone (QVD) was obtained from Bio‐Connect (#A1901).

### 2.3 KiNativ™ assay

Cells were grown to confluence in appropriate media and treated with indicated concentrations in 0.1% dimethyl sulfoxide (DMSO) for 1 h. Cells were rinsed once with ice‐cold PBS, scraped off, and transferred to a 15 mL conical tube and pelleted by centrifugation and stored at −80 °C. KiNativ profiling of protein and lipid kinases was performed by ActivX Biosciences (La Jolla, CA, USA). Briefly, lysates were prepared from cell pellets and incubated with ADP and ATP probes. After a tryptic digest, the probe‐labeled peptides were characterized and quantified using targeted liquid chromatography–mass spectrometry (LC–MS). Comparison of MS signals from treated and untreated cells identified kinases bound by the compounds.

### 2.4 Generation of CRISPR/Cas 9 knockouts

BAK, BAX, BID, BIM, NOXA, and PUMA knockout cells were generated using CRISPR/Cas9 technology. Single‐guide RNAs (sgRNAs) were selected using Deskgen (deskgen.com) or from literature [22]. Each sgRNA was cloned in the lentiCRISPRv2 puro plasmid, a gift from Brett Stringer (Addgene #98290; Watertown, MA, USA). Lentivirus was produced and used to infect the cell lines. After selection with puromycin (Sigma #P8833), knockouts were confirmed by SDS/PAGE. sgRNAs used are listed in Table S1.

### 2.5 Cell Titer‐Glo experiments

For the majority of lines, dose–response data were generated by spotting increasing concentrations of compound (1.5 nm to 10 μm) via an acoustic dispenser (EDCATS‐100) into an empty 384‐well plate. Compound was spotted in a 10‐point serial dilution fashion (3‐fold dilution) in duplicate within the plate. The DMSO concentration was kept constant for a final assay concentration of 0.1% DMSO. For testing, cells were diluted to the appropriate densities and added directly to the compound‐spotted 384‐well plates. At the time when compound was added (t0), initial cell number was assessed using Cell Titer‐Glo (Promega, Madison, WI, USA) by quantifying the level of luminescence generated by ATP present in viable cells. After 72 h, the cell viability of compound‐treated cells was assessed using Cell Titer‐Glo. Percent growth was calculated using the formula: (72 h luminescence − t0 luminescence)/t0 luminescence × 100. Data were plotted and analyzed in graphpad prism (Graphpad Software, San Diego, CA, USA). Results were expressed as GI50 and Emax values. GI50 is the compound concentration required to inhibit cell growth in treated cells to 50% of the growth of the untreated control cells. Emax is the percent growth at 10 μm compound (the maximum concentration of compound used in the assay).

For a minority of cell lines, dose–response data were generated by using manual dilution series of compound instead of using an acoustic dispenser, and working in a 96 instead of a 384‐well format, but otherwise, the protocols were the same.

### 2.6 Protein lysate preparation

Cell pellets were resuspended in ice‐cold Cell Extraction Buffer (Thermo Fisher Scientific #FNN0011; Waltham, MA, USA) supplemented with 1× Halt Protease Inhibitor Cocktail (Thermo Fisher Scientific #87785) or lysed in RIPA sample buffer (150 mm NaCL, 1 mm EDTA, 50 mm Tris–HCL pH 7.4). Pellets were sonicated with two 5 s bursts under low amplitude (20%) using the Fisher Scientific 150E Digital Sonic Dismembrator. Xenograft samples were homogenized using the Retsch Mixer Mill MM 400 prior to sonication. Lysates were clarified by centrifugation at 4 °C. Protein concentrations were measured by bicinchoninic acid (BCA) assay (Thermo Fisher Scientific #23225). Sample protein concentrations were normalized in 1× NuPAGE LDS Sample Buffer (Thermo Fisher Scientific #NP0007) plus 1× NuPAGE Sample Reducing Agent (Thermo Fisher Scientific #NP0009). Samples were denatured by heating to 80 °C for 10 min.

### 2.7 SDS/PAGE and western blot

Equal amounts of protein were subjected to SDS/PAGE using 3–8% tris‐acetate protein gels (Thermo Fisher Scientific #A03755BOX, for DNA‐PK, and vinculin detection) and 4–12% bis‐tris protein gels (Thermo Fisher Scientific #NP0323BOX, for detection of other proteins). Proteins were electrophoretically transferred to 0.2 μm nitrocellulose membranes (Thermo Fisher Scientific #LC2000) and blocked for 1 h with Odyssey Blocking Buffer TBS (LI‐COR #927‐50000) or 2% bovine milk. Membranes were incubated overnight with primary antibody and secondary antibody for 1 h. Membranes were washed with TBST/TBS and scanned on the Odyssey Infrared Imager (LI‐COR). Antibodies used in this study are listed in Table S2.

### 2.8 Real‐Time PCR and NMD assay

Total RNA was prepared using Qiagen's RNeasy Mini kit (#74104; Germantown, MD, USA) or GeneEluteTM MammalianTotal RNA Miniprep kit (Sigma‐Aldrich #RTN70) following the manufacturer's instructions. Contaminating gDNA was eliminated by DNase digestion. RNA was reverse transcribed into cDNA using Applied Biosystem's High Capacity RNA‐to‐cDNA Kit (#4387406; San Francisco, CA, USA) or by the M‐MuLV Reverse Transcription System (Fermentas Inc., Hanover, MD, USA #EP0451) using Random Hexamer Primers (Promega #C1181). cDNA was diluted 10× with nuclease‐free H2O and mixed with a final concentration of 200 nm forward and reverse primers. Fast SYBR Green master mix (Applied Biosystems #4385617) or 100 nm Taqman probe, 1× Taqman PCR master mix was used according to the Applied Biosystem protocols. *GAPDH* and *HPRT1* were used as housekeeping genes. linreg software was used for data processing [23]. Relative expression was calculated by the comparative Δ*C*_t_ method. In indicated experiments, qPCR results were normalized to control gene *HPRT1* and relative to DMSO. Primers for NMD transcripts were designed around exons that were normally subjected to NMD, using two primer pairs that would detect either the NMD exon or as a control the two bordering exons. Primers used in this study are listed in Table S3.

### 2.9 Cell death assays

Cells were seeded in 96‐well plates (40 000 cells per well) and incubated in the presence of different compounds for indicated time points. Viability was measured by 0.01 μm Dihexyloxacarbocyanine Iodide (DiOC6, Molecular Probes #D‐273; Eugene, OR, USA) after incubation for 20 min at 37 °C. Prior to analysis, 5 nm TOPRO‐3 Iodide (Thermofisher Scientific #T3605) was added. Cryopreserved bone marrow mononuclear cells of MM patients were thawed and rested o/n in the presence of 10 ng·mL^−1^ rhIL‐6. The next day, the cells were seeded at 100 000 cells per well in a 96‐well plate with 10 ng·mL^−1^ rhIL‐6 and treated at indicated time points and stained with CD38 (BD Biosciences #345807; San Jose, CA, USA), CD138 (BD Biosciences #341107), and Annexin V‐EGFP (IQ Products; #iqp‐120f; Houston, TX, USA). Prior to analysis, propidium iodide (Sigma #P4864; PI) was added. The average viability of the CD38^+^/CD138^+^ cells at the time of analysis was 74.7%. Specific apoptosis was defined as ([% cell death in treated cells] − [% cell death in medium control])/[% viable cells medium control] × 100.

### 2.10 *In vivo* studies

All animal studies were performed under protocols approved by Institutional Animal Care and Use Committees. Animals were housed and xenograft studies were performed as previously published [12]. All animal studies were performed under protocols approved by the Celgene Institutional Animal Care and Use Committee (IACUC). Female 6‐ to 8‐week‐old CB17 SCID (severe combined immunodeficiency; Charles River Laboratories, Wilmington, MA, USA) mice were housed in a barrier facility in micro‐isolator cages at 10 animals per cage. Mice were fed with Harlan‐Teklad LM‐485 Mouse/Rat Sterilizable Diet and autoclaved water *ad libitum* and maintained on a 12‐h light/dark cycle. All animals in the study were tagged with stainless steel metal ear tags. Animals were acclimatized to the animal housing facility for a period of 7 days prior to the beginning of the experiment.

Suspensions of CC‐223 were prepared in aqueous 0.5% carboxymethyl cellulose and 0.25% Tween‐80. The formulations were homogenized using a Teflon pestle and mortar (Potter‐Elvehjem tissue grinder). For multiday studies, the compound was freshly formulated every third day. Between doses, the formulated compound was stored under constant stirring using a magnetic stirrer at 4 °C in the dark. The test article and vehicle were administered by oral gavage.

SCID mice were inoculated subcutaneously with 10 × 10^6^ ANBL‐6 cells. When tumors reached approximately 75–150 mm^3^, mice were randomized into various treatment groups and administered PO with CC‐223 or CC‐115 once daily at a dose volume of 5 mL·kg^−1^. Tumor volumes were determined prior to the initiation of treatment and were considered as the starting volumes. Tumors were measured twice a week for the duration of the study. The long and short axes of each tumor were measured using a digital caliper in millimeters. The tumor volumes were calculated using the formula: width^2^ × length/2. The tumor volumes were expressed in cubic millimeters (mm^3^).

Xenograft data are expressed as mean ± SEM. Statistical analyses were performed using graphpad prism. A one‐way analysis of variance (ANOVA) was performed for tumor volume measurements. *Post‐hoc* analysis was performed using Dunnett's test where all treatment groups are compared with the vehicle control.

The study was designed to compare the antitumor activity of CC‐223 and CC‐115. Doses of 10 mg·kg^−1^ for CC‐223 and 5 mg kg^−1^ for CC‐115 were selected to give similar plasma exposure to both agents [12, 13, 24]. Dosing started on day 8 when tumor volumes ranged between 75 and 150 mm^3^ and continued until day 36.

### 2.11 Statistical analyses

One‐way ANOVA, with the Greenhouse–Geisser correction and Dunnett's *post‐hoc* test was applied to analyze differences between more than two groups. Mann–Whitney *U*‐test was used to analyze differences between the two groups. A *P*‐value < 0.05 was considered statistically significant.

## 3 Results

### 3.1 Identification of SMG1 as an additional target of CC‐115

**Fig. 1:**
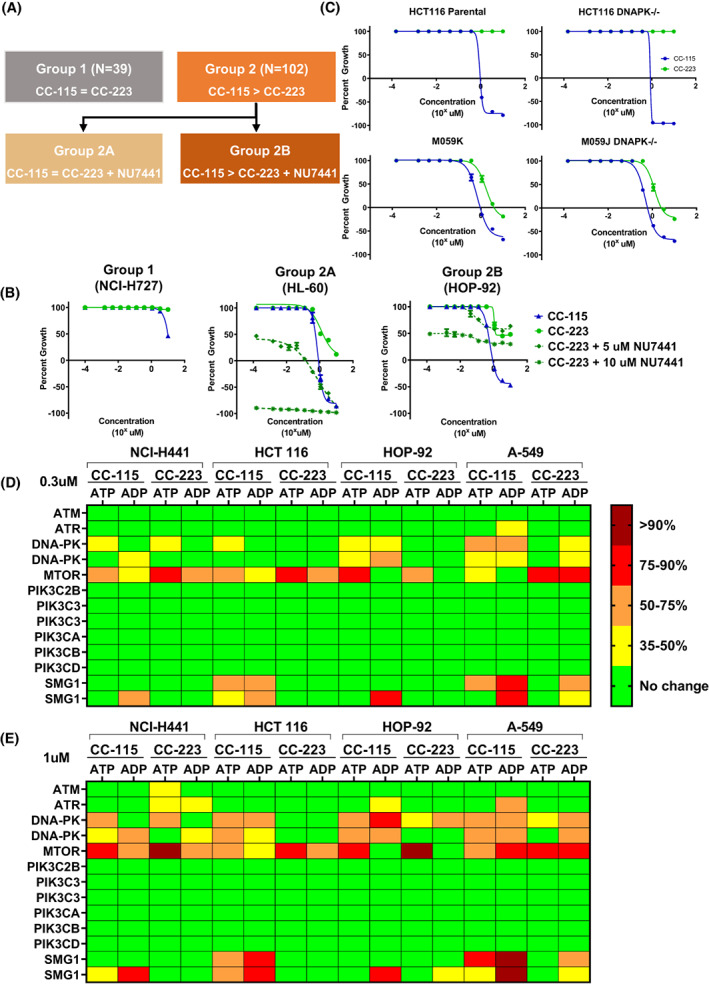
CC‐115 has another target, beyond TORK and DNA‐PK. (A) Schematic of cell line grouping based on cellular response to TORK inhibitors CC‐223 or CC‐115 (Table S1). For a subset of cell lines at Emax (the percent growth at 10 μm compound), CC‐115 activity was greater than CC‐223, and CC‐115 activity was greater than that of an inhibitor of TORK (CC‐223) plus an inhibitor of DNA‐PK (NU7441). (B) Dose–response growth curves of cell lines representative of groups 1, 2A, and 2B after 72‐h incubation with increasing concentrations of compound (1.5 nm to 10 μm). Depending on the growth speed of the cell lines, seeding density was either 2000 or 5000 cells per well. Cell viability of control and compound‐treated cells was assessed using cell titer‐Glo. Error bars are ±SEM of three independent experiments. (C) Dose–response growth curves for isogenic DNA‐PK knockout cell lines HCT 116/HCT 116 DNA‐PK^−/−^ and M059K/M059J DNA‐PK^−/−^ treated with CC‐115 or CC‐223. Error bars are ±SEM of three independent experiments. (D, E) Results from ActivX KiNativ analysis comparing different doses of CC‐115 or CC‐223 in four different group 2B cell lines. Results for (D) 0.3 μm dose or (E) 1.0 μm dose. Treated samples were in duplicate; control samples were in duplicate or quadruplicate. Color coding refers to the amount of competitive binding observed (dark red > 90%, red 75–90%, orange 50–75%, yellow 35–50%, and green no change or peptide was not detected for the particular proteome/probe). The percent changes in mass spectrometry signals being reported are statistically significant (student's *t*‐test *P* < 0.04). Results for the total KiNativ dataset including all other kinases are shown in Table S5.

Preliminary testing had indicated that CC‐115, described previously as an inhibitor of mTOR kinase (TORK) and DNA‐PK, [12, 13, 14] may have additional specific target(s). Screening of a panel of 141 cancer cell lines identified stronger activity of CC‐115 in a subset, compared to the specific TORK inhibitor CC‐223 [24] (Table S4). To test the involvement of DNA‐PK, cell lines were treated with CC‐223 (alone or in combination with the established DNA‐PK inhibitor NU7441) or with CC‐115 alone, and grouped based on differential sensitivity (Fig. 1A,B). In several cell lines, dual inhibition of TORK and DNA‐PK by CC‐115 [13, 15, 16] could not fully explain the activity of CC‐115. The effect of CC‐115 on cell growth was greater than that of CC‐223 in numerous cell lines, including isogenic DNA‐PK^−/−^ cell lines (HCT 116 and M059J; Fig. 1C), indicating that CC‐115 indeed had additional target(s) beyond TORK and DNA‐PK. In order to identify other target(s), CC‐115‐ or CC‐223‐treated cell lysates were analyzed in the ActivX KiNativ platform [25]. Evaluation of > 200 kinases confirmed inhibition of TORK (FRAP) and DNA‐PK by CC‐115 and identified SMG1 as an additional target of CC‐115 (Fig. 1D,E and Table S5).

### 3.2 Multiple myeloma cells are sensitive to SMG1 kinase inhibition

**Fig. 2:**
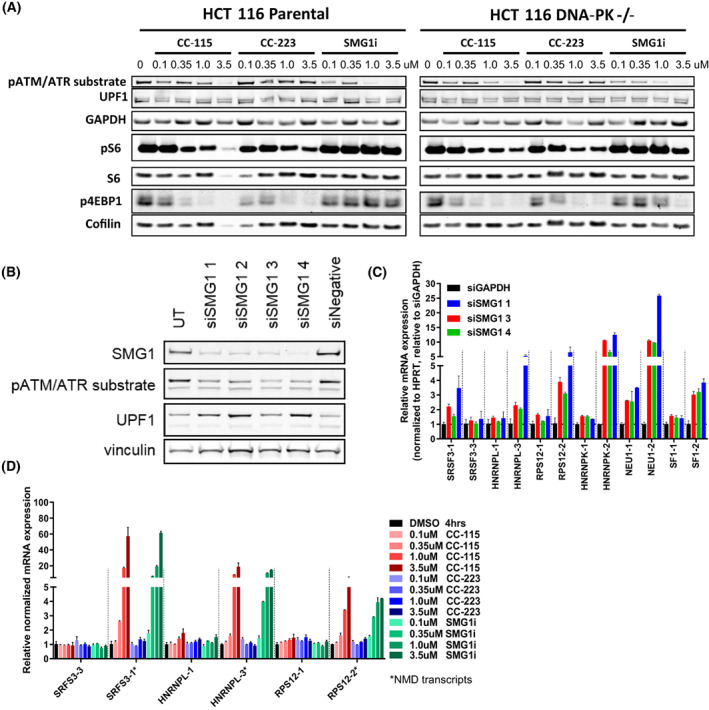
CC‐115 is an inhibitor of SMG1‐mediated NMD. (A) Western blot analysis for markers of TORK inhibition (pS6 and p4EBP1) and SMG1 inhibition (pATM/ATR substrate band colocalizing with UPF1) in isogenic HCT 116 cell lines (parental and DNA‐PK^−/−^). Treatment time for this western was 4 h. cofilin is a low MW loading control. The reduction of cofilin in the 3.5 μm CC‐115 treated HCT 116 parental is due to less protein being loaded in this lane. Total UPF1 and GAPDH were run on a different gel. (B) SMG1 western blot confirming siRNA knockdown of SMG1 and reduction in phospho‐UPF1 in HCT‐116 parental cells. (C) qPCR analysis of NMD transcripts in siSMG1 transfected HCT‐116 parental cells (qPCR to normalized control gene HPRT1 and relative to siGAPDH). Error bars are ±SEM of two independent experiments. * = NMD. (D) CC‐115 or SMG1i, but not CC‐223, induced the expression of NMD transcripts in a dose‐dependent manner in HCT 116 parental cells at 4 h after treatment (qPCR normalized to control gene HPRT1 and relative to DMSO). Error bars are ±SEM of two independent experiments. * = NMD transcript.

**Fig. 3:**
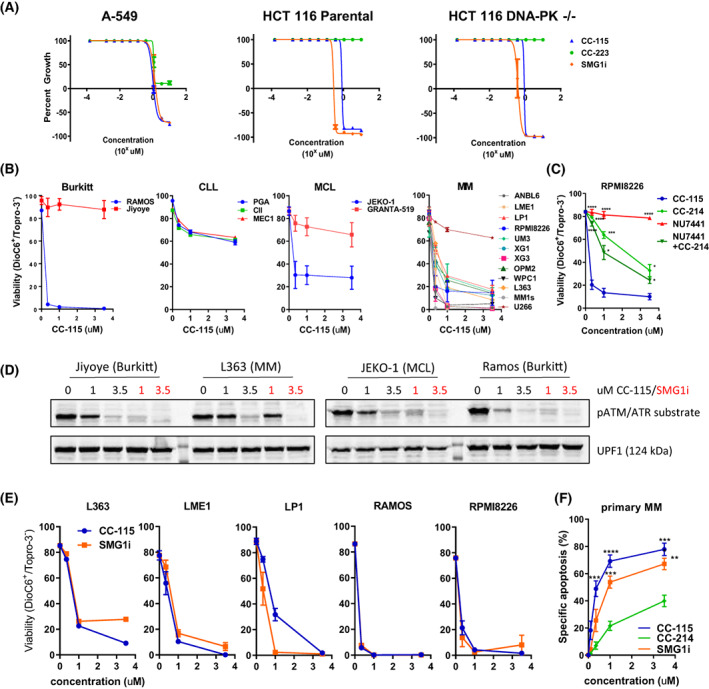
Multiple myeloma cells are sensitive to SMG1 kinase inhibition. (A) 50% growth inhibition (GI50) curves for a group 2B cell line (A‐549) and isogenic HCT 116 cell lines (parental and DNA‐PK^−/−^) treated with CC‐115, CC‐223, or SMG1i for 72 h with increasing concentrations of compound (1.5 nm to 10 μm). Error bars are ±SEM of three independent experiments. (B) Effect of CC‐115 on viability (48 h) measured by flow cytometry with DIOC6/TOPRO‐3 staining in cell lines from B‐cell malignancies: Burkitt's lymphoma, chronic lymphocytic leukemia (CLL), mantle cell lymphoma (MCL), and multiple myeloma (MM). Error bars are ±SEM of three independent experiments (BL and CLL) or two independent experiments (MM). (C) Representative MM cell line (RPMI8226) in which CC‐115 had more activity than the combination of a TORK inhibitor (CC214‐1) with a DNA‐PK inhibitor (NU7441). Cells were treated for 48 h and stained with DIOC6/TOPRO‐3. Shown is the mean ± SEM of five biologically independent experiments, one‐way ANOVA with Dunnett's *post‐hoc* test compared to CC‐115. (D) Western blot analysis for SMG1 inhibition (pATM/ATR substrate band colocalizing with UPF1) in various B cell lines, after 16‐h treatment with indicated concentrations of CC‐115 or SMG1i. (E) Effect of SMG1 inhibition by SMG1i on viability (48 h) measured by DIOC6/TOPRO‐3 staining in comparison with CC‐115 in five different cell lines. Shown is the mean ± SEM of two independent experiments. (F) Effect of CC‐115 or CC214‐1 on specific apoptosis in primary MM cells (*N* = 7). Cells were treated for 72 h and stained with AnnexinV/PI. Specific apoptosis is plotted to correct for variability in background apoptosis in these clinical samples. Error bars are ±SEM, one‐way ANOVA with Dunnett's *post‐hoc* test compared to CC214‐1, **P < 0.01, ***P < 0.001, ****P < 0.0001.

Similar to other PIKK, SMG1 can phosphorylate serine (S) or threonine (T) residues in the context of the SQ/TQ motif [20, 26]. SQ/TQ phosphorylation of UPF1 was decreased in a dose‐dependent manner by CC‐115 and also by another SMG1 inhibitor (SMG1i), but not by CC‐223 in HCT 116 parental and HCT 116 DNA‐PK^−/−^ cells (Fig. 2A). SMG1i had no effect on bleomycin‐induced DNA‐PK phosphorylation (Fig. S1) or TORK (using downstream markers phosphorylation of S6 and phosphorylation of 4EBP1; Fig. 2A). As expected, knockdown of SMG1 decreased phosphorylation of UPF1 and showed an increase of NMD‐sensitive transcripts (Fig. 2B,C). Both CC‐115 and SMG1i, but not CC‐223, caused a dose‐dependent increase in the expression of NMD transcripts (Fig. 2D). To confirm that cell death correlated with SMG1 inhibition, A549 and isogenic HCT 116 cell lines (parental and DNA‐PK^−/−^) were treated with CC‐115, CC‐223, and SMG1i. In these cell lines, SMG1i blocked proliferation and survival to the same extent as CC‐115 (Fig. 3A).

We tested the effects of CC‐115 and SMG1i on various human cancer cell lines, focusing on hematological malignancies, as Kinome‐wide RNAi studies included SMG1 in a list of active kinases in MM models [27]. Differential responses were found in Burkitt's lymphoma (BL), chronic lymphocytic leukemia (CLL), mantle cell lymphoma (MCL), and multiple myeloma (MM). These analyses demonstrated that MM cell lines and the BL cell line RAMOS were particularly sensitive to CC‐115 (Fig. 3B). Burkitt or MCL cell lines displayed both sensitive or resistant phenotypes, while CLL cell lines PGA, CII, or MEC‐1 were all relatively resistant. In an extension of previous work on primary CLL cells (Thijssen et al. [15]), we tested whether CC‐115 was synergistic with the Bcl‐2 inhibitor Venetoclax in these cell lines, which was indeed the case (Fig. S2). This suggests that these cell lines need Bcl‐2 to protect them against CC‐115‐induced cell death. Comparison of CC‐115 vs. specific inhibitors of TORK and DNA‐PK confirmed that cell death induction was predominantly due to SMG1 inhibition (Fig. 3C and Fig. S3). Effects of CC‐115 and SMG1i on SMG1 were measured by examining pUPF1, using phosphorylation of pATM/ATR substrate as in Fig. 2A. As shown in Fig. 3D, in insensitive (Jiyoye) as well as sensitive (L363, JEKO‐1, Ramos) cell lines both inhibitors blocked UPF‐1 phosphorylation. This indicates that although inhibition of UPF phosphorylation was apparent in all lines tested, this does not necessarily lead to cell death. Cell death induction by CC‐115 or the more specific SMG1 inhibitor was comparable in all cell lines tested (Fig. 3E). Importantly, primary MM cells from patients (*n* = 7) were also highly sensitive to CC‐115 and the more specific SMG1 inhibitor (Fig. 3F).

### 3.3 Inhibition of SMG1 induces UPR transcripts and apoptosis in MM cells

MM cells are responsive to ER stress, which then activates different branches of the unfolded protein response (UPR) [28]. Recent studies have described an interplay between the UPR and NMD [7]. Therefore, involvement of the UPR, or similarity in terms of responses, appeared logical, and we explored a possible overlap or correlation in response pathway(s).

**Fig. 4:**
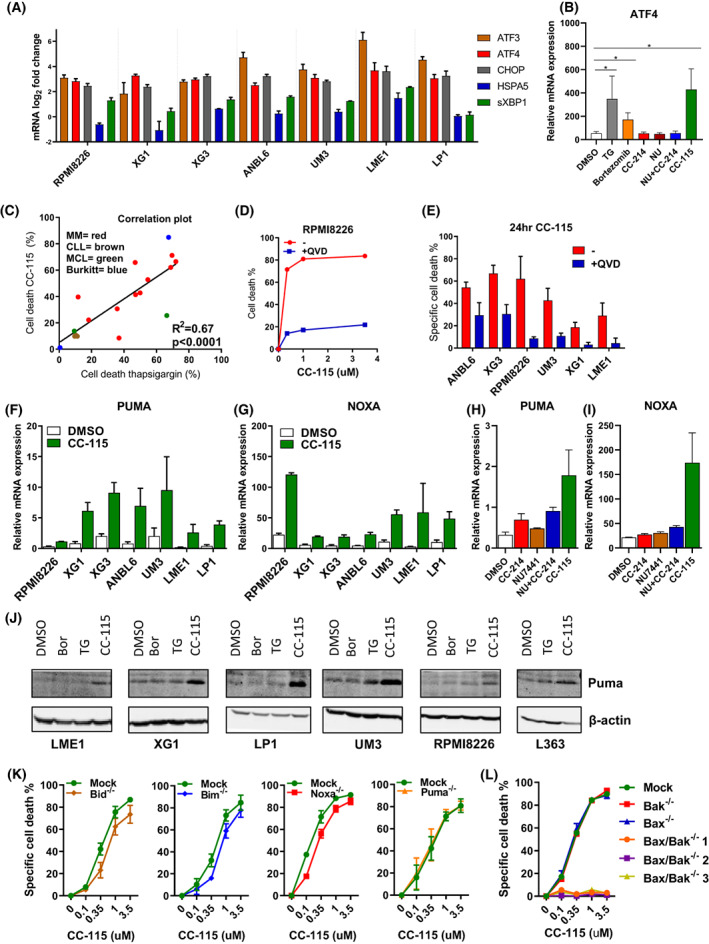
CC‐115 treatment induces UPR transcripts and apoptosis in multiple myeloma cell lines. (A) Bar plots representing the log2 fold change of *ATF3*, *ATF4*, *CHOP*, *HSPA5*, and *sXBP1* mRNA levels (determined by qPCR; ΔΔ*C*_t_ with DMSO treatment as control) after 16‐h incubation with CC‐115 (1 μm) in the presence of quinoline‐Val‐asp‐Difluorophenoxymethylketone (QVD; 5 μm) with *GAPDH* as reference gene. Shown is the mean ± SEM of two independent experiments. (B) Effect of CC‐115 on ATF4 mRNA expression in comparison with TORK and/or DNA‐PK inhibition. Cells were incubated in the presence of the UPR inducing agents bortezomib (20 nm), thapsigargin (TG; 20 nm), TORK inhibition by CC214‐1 (1 μm), DNA‐PK inhibition by NU7441 (1 μm), the combination NU7441 (1 μm) + CC214‐1 (1 μm), CC‐115 (1 μm), or equal concentration of DMSO. Bars represent relative mRNA expression of four independent experiments and error bars represent SD, one‐way ANOVA with Dunnett's *post‐hoc* test compared to DMSO, *P < 0.05, ***P < 0.001. (C) Correlation plot of cell death induced by CC‐115 (1 μm) vs. cell death induced by thapsigargin (100 nm; 24 h) for cell lines used in Fig. 3B. Correlation was determined by a spearman rho test. (D) Effect of QVD treatment on cell death after 48‐h treatment with CC‐115 in MM cell line RPMI8226 measured by flow cytometry via DIOC6/TOPRO‐3 staining. Shown is the mean ± SEM of three experiments. (E) Effect of QVD (5 μm) on cell death induced by 0.35 μm CC‐115 (24 h) in different MM cell lines. Cell death was calculated via difference in viability (DIOC6^+^/TOPRO‐3^−^) between DMSO and drug treatment. Shown is the mean ± SEM of two independent experiments. (F–I) Bar plots representing the effects of CC‐115 (16 h; 1 μm) on *PUMA* (F) and *NOXA* transcription (G) and the effect of CC214‐1 (1 μm), NU7441 (1 μm), NU7441 (1 μm) + CC214‐1 (1 μm), CC‐115 (1 μm), or equal concentrations of DMSO on *PUMA* (H) and *NOXA* transcription (I) determined by qPCR. For qPCR experiments, cells were incubated in the presence of QVD (5 μm) and *GAPDH* was used as housekeeping gene. Shown is the mean ± SEM of two independent experiments. (J) Effect of bortezomib (Bor; 1 μm), thapsigargin (TG; 3 μm), or CC‐115 (16 h; 1 μm) on PUMA determined by western blotting in six different MM cell lines. (K, L) Effect of CRISPR/Cas9 knockout of *BID*, *BIM*, *NOXA*, *PUMA* (K), *BAK*, *BAX*, and *BAX/BAK* double knockout (L), on cell death by CC‐115 (24 h) in RPMI8226 cells measured by flow cytometry via DIOC6/TOPRO‐3 staining. Shown is the mean ± SEM of seven independent experiments for mock, *BAK*, and *BAX* knockout cell lines and four independent experiments for other cell lines.

CC‐115 clearly increased RNA levels of *ATF4*, *ATF3*, and *CHOP*, but not so much *HSPA5* or *sXBP1* across the MM cell line panel. These effects were due to SMG1 inhibitory activity, as *ATF4*, *ATF3*, and *CHOP* were upregulated by CC‐115, but not by the TORK inhibitor CC214‐1 (Fig. 4A and Fig. S4). As a positive control, MM cell lines were exposed to established inducers of ER stress, such as thapsigargin (TG) and bortezomib, which induced a similar upregulation of *ATF4*, *ATF3*, and *CHOP* (Fig. 4B). Sensitivity to CC‐115 correlated with TG sensitivity across cell lines (Fig. 4C).

Cell death via CC‐115 was inhibited by the pan‐caspase inhibitor QVD (Fig. 4D,E), indicating that cell death occurred via caspase‐mediated apoptosis. CC‐115 treatment resulted in increased expression of the pro‐apoptotic genes *PUMA* and *NOXA* (Fig. 4F–I). An increase in Noxa protein in some but not all cell lines could be observed (data not shown). A discrepancy between Noxa transcript and protein levels has been noted before and was ascribed to its rapid protein turnover [29, 30]. In contrast, for PUMA, a consistent protein increase across the cell lines was clear (Fig. 4J). Various BH3‐only members have been implicated in cell death by ER stress [28, 31, 32]. Single knockout (KO) of *BID*, *BIM*, or *NOXA* by CRISPR/Cas9 each modestly decreased sensitivity to CC‐115, while deletion of *PUMA*, *BAK*, or *BAX* had no effect (Fig. 4K,L and Fig. S5). In contrast, KO of BAX and BAK together completely prevented CC‐115‐induced death (Fig. 4L **)**.

In conclusion, MM cells are particularly sensitive to SMG1 inhibition by CC‐115 and SMG1i, which causes an integrated stress response and apoptosis.

### 3.4 CC‐115 inhibits SMG1 kinase *in vivo* and blocks MM xenograft tumor growth

**Fig. 5:**
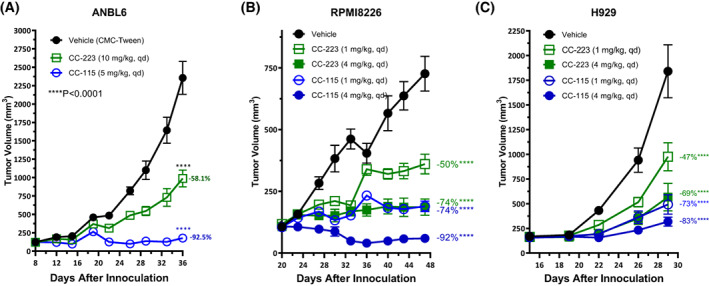
CC‐115 inhibits MM tumor growth *in vivo*. (A) Antitumor activity of CC‐223 and CC‐115 in multiple myeloma xenograft model with once daily (QD) dosing. Groups of (*n* = 10 per each treatment group) mice with ANBL6 tumors were treated PO with vehicle, CC‐223 or CC‐115. (B, C) similar experiments with RPMI8226 and H929 cell lines using two doses of CC‐115 and CC‐223 (*n* = 9 mice per group). Each data point represents the mean tumor volume of each treatment group. Error bars are ±SEM. *P*‐values were calculated by using one‐way ANOVA with Dunnett's *post‐hoc* test compared to vehicle at end of the study.

Effects of SMG1 inhibition were tested in *in vivo* models, using CC‐115 as it has already been tested in humans in advanced solid and hematologic malignancies [15], and SMGi compound cannot be applied *in vivo*. The ability of CC‐115 to inhibit SMG1 and thus NMD *in vivo* was confirmed in an early stage of the project by qPCR analysis of HCT 116 xenograft tumors after treatment of mice with CC‐115 (Fig. S6). The effect of CC‐115 vs. TORK inhibition was tested in three MM xenograft models, including the ANBL6 cell line, which is transcriptionally similar to patient MM samples [33]. In mice treated with CC‐115 tumors were 177 ± 25 mm^3^ at the end of the study (*P* < 0.0001), close to their starting tumor volume of 75–150 mm^3^. In contrast, treatment with the TORKi, CC‐223, decreased tumor size ~2× relative to vehicle‐treated mice (987 ± 114 vs. 2354 ± 225 mm^3^, respectively, Fig. 5A). The widely used RPMI8226 cell line was tested at two doses of CC‐115 and CC‐223 and also showed clear tumor volume reduction, to below starting volume for 4 mg·kg^−1^ CC‐115 (Fig. 5B). H929 cells also showed a statistically significant higher reduction in tumor volume after treatment with CC‐115 (Fig. 5C). Of note, the cell lines showed intrinsic differences in response to the drugs, yet the advantage of CC‐115 over CC‐223 remained clear. In all cases, CC115 was well tolerated, as body weight was not affected (Fig. S7). In conclusion, CC‐115 showed superior efficacy over CC‐223 and was well tolerated in three MM *in vivo* models.

## 4 Discussion

In the present study, we identified SMG1 as a novel target of CC‐115, in addition to its known targets, TORK and DNA‐PK. Inhibition of SMG1, independent of TORK and DNA‐PK, appeared to be the prime mode of CC‐115‐induced caspase and BAX‐ and BAK‐dependent cell death in certain tumor cells.

Among B‐cell malignancies, MM appeared exquisitely sensitive to SMG1 inhibition. In line with our results, a Kinome‐wide RNAi screen in MM cells listed SMG1 as a promising novel target [27]. The serine/threonine kinase AKT is upregulated in MM [34] and active AKT signaling has been linked to hyperactivation of NMD [35], indicating the dependency of MM cells on NMD activity. Moreover, whole genome sequencing identified UPR proteins as frequently mutated in MM [36] and plasma cells, which are characterized by high protein secretion, rely on the UPR for survival [37].

Previous reports indicate that NMD and UPR are functionally linked, showing that NMD prevents the activation of UPR by increasing the threshold for apoptosis [5, 6, 8, 31]. UPR activation via SMG1 inhibition displayed similarities with TG and bortezomib. CC‐115 and TG increased *ATF4* mRNA, which fits with the observation that inhibition of NMD stabilizes *ATF3* and *ATF4* transcripts [7, 38]. An aspect which requires further investigation is that despite clear indications that cell death by CC‐115 occurs by the mitochondrial pathway of apoptosis, and correlates with induction of Puma and/or Noxa expression, we could not pinpoint a specific BH3‐only protein to be responsible. In a recent study, GSPT1 degradation by new thalidomide analogs resulted in an activated integrated stress response and *TP53*‐independent cell death [39]. Similarly, we could not correlate TP53 mutation status with sensitivity to SMG1 inhibition, suggesting clinical activity of SMG1 inhibition in high‐risk cancers. Future studies beyond the scope of the current article are directed toward further elucidating the cell death mechanism, and the differences between CC‐115 sensitive and resistant cell lines in terms of cell death.

The observed *in vivo* efficacy and tolerability of CC‐115 in three MM xenotransplant models raises the possibility that CC‐115 could have clinical efficacy with limited toxicity. Earlier studies of CC‐115 were performed under the assumption that DNA‐PK inhibition by CC‐115 is synthetically lethal in ATM‐deficient cells [16]. Phase I trials were enriched for CLL patients with del(11q)/ATM defects and no MM patients were included [15, 16, 40]. In our hands, CLL cell lines were relatively insensitive and seemed to be protected by high BCL‐2 expression, as combination therapy of CC‐115 with the BCL‐2 inhibitor venetoclax showed synergy in three investigated CLL cell lines (Fig. S2).

## 5 Conclusion

Based on current knowledge, MM might be further exploited for the clinical treatment of cancer with SMG1 inhibitors. In addition to cell‐autonomous effects of inhibition of SMG1/NMD, inhibition of NMD may increase tumor antigens and immunogenicity of tumors, thereby potentially stimulating an antitumor immune response [4, 41, 42]. SMG1 inhibition can increase activation of the innate immune response via its effect on the maturation of dendritic cells [43]. Thus, rational combinations for an SMG1 inhibitor might include immunomodulatory agents and T‐cell activating drugs such as immune checkpoint inhibitors. Furthermore, SMG1 inhibitors might be effective in other malignancies that depend on the UPR for survival such as Waldenström's macroglobulinemia, acute myeloid leukemia (AML), as well as other solid tumor types.

## Conflict of interest

This work was sponsored via a research agreement between Amsterdam University Medical Centers and Bristol Myers Squibb. BG, MH, MM, DM, KB, MC, JCL, MCG, RKN, RL, TT, and EHF were/are employees of Bristol Myers Squibb. The remaining authors declare no conflict of interest.

## Author contributions

ACL, APK, EE, and EHF were responsible for the conception and design of studies. ACL, BG, TT, JCL, TB, M‐JK, MH, MM, MCG, RKN, JG, AJ, MC, RL, PDM, APK, EE, and EHF were involved in analysis and interpretation of data and contributing text. ACL, AJ, and PDM performed statistical analyses. ACL, IAMD, MCG, DSM, KB, JCL, MC, MM, MH, RKN, TT, EHF, and BG performed experiments, and ACL, IAMD, MC, MM, MH, RKN, TT, EHF, and BG made figures.

### Peer review

The peer review history for this article is available at https://publons.com/publon/10.1002/1878‐0261.13343.

## Supporting information


**Table S1.** Single guide RNAs used for generation of different CRISPR/Cas9 knockout cells.
**Table S2.** Antibodies used for western blotting analysis.
**Table S3.** Primers used for Real‐Time quantitative PCR.
**Table S4.** GI50 and Emax for 141 cell lines in a 3‐day proliferation assay treated with CC‐115 and CC‐223.
**Table S5.** Results from ActivX KiNativ analysis comparing different doses of CC‐115 or CC‐223 in four different Group 2B cell lines.
**Fig. S1.** SMG1i does not inhibit the Ser2056 phosphorylation of DNA‐PK.
**Fig. S2.** Block titration of CC‐115 plus ABT‐199 in three CLL cell lines.
**Fig. S3.** Effect of CC‐115 on viability in comparison with DNA‐PK and/or TORK inhibition in MM cell lines.
**Fig. S4.** Effect of CC‐115 on UPR‐related transcripts.
**Fig. S5.** Validation of different KO cell lines by western blotting.
**Fig. S6.** qPCR analysis on HCT 116 xenograft tumors treated with Vehicle or CC‐115 (qPCR normalized to control gene HPRT1 and relative to vehicle).
**Fig. S7.** Bodyweight monitoring in *in vivo* mouse experiments, related to Fig. 5.

## Data Availability

The original contributions presented in the study are included in the article/supplementary material, further inquiries can be directed to the corresponding author/s.
