# Supplementary material for: SMG1, a nonsense‐mediated mRNA decay (NMD) regulator, as a candidate therapeutic target in multiple myeloma
Source: Mol Oncol. 2022 Dec 16;17(2):284–97. doi: 10.1002/1878-0261.13343 (PMC9892823; doi:10.1002/1878-0261.13343)

Table S1: Single guide RNAs used for generation of different CRISPR/Cas9 knock-out cells

Gene	sgRNA sequence	Exon	Source
Bid	GCTCATCGTAGCCCTCCCAC	3	O'Neil et al, Genes Dev., 2016
Bim	GTAAGATAACCATTCGTGGG	5	Deskgen.com
Noxa	TCGAGTGTGCTACTCAACTC	2	O'Neil et al, Genes Dev., 2016
Puma	TCAACGCACAGTACGAGCGG	3	Deskgen.com
Bak	ACGGCAGCTCGCCATCATCG	5	O'Neil et al, Genes Dev., 2016
Bax	CTGCAGGATGATTGCCGCCG	4	O'Neil et al, Genes Dev., 2016
ATF4	ATCACAAGTGTCATCCAACG	2	Deskgen.com

Table S2: Antibodies used for western blotting analysis

Antibody	Company/Catalog No.	Dilution
DNA-PK	Cell Signaling #12311	1:1000
phospho DNA-PK (Ser2056)	Abcam #ab124918	1:1000
vinculin	Sigma #V9264	1:10.000
phospho-(Ser/Thr) ATM/ATR substrate	Cell Signaling #2851	1:1000
UPF1	Sigma #SAB1402893	1:2000
GAPDH	Santa Cruz #47724	1:10.000
total S6 Ribosomal Protein	Santa Cruz #74459	1:5000
phosphoS6 Ribosomal Protein (Ser240/244)	Cell Signaling #5364	1:1000
phospho-4E-BP1 (Thr70)	Cell Signaling #9455	1:1000
BAK	Cell Signaling #12105S	1:1000
BAX	Cell Signaling #5023S	1:1000
BIM	StressMarq #SPC-113	1:500
BID	Cell Signaling #2002	1:1000
Noxa	Novus Biologicals #NB600-1159	1:500
Puma	Sigma-Aldrich #PRS3043	1:500
Caspase-8	Santa Cruz #sc-6136	1:1000
actin	Santa Cruz #sc-1616	1:4000
cofilin	Santa Cruz #37647	1:10.000
IRDye 800CW Goat anti-Rabbit IgG	LI-COR #926-32211	1:15.000
IRDye 800CW Donkey anti-Goat	LI-COR #926-32214	1:15.000
IRDye 680LT Donkey anti-Goat	LI-COR #926-32224	1:20.000
IRDye 680LT Goat anti-Mouse IgG	LI-COR #926-68020	1:20.000

Table S3: Primer sequences used for qPCR analyses

Gene	Primer name	sequence	Notes
SRSF3	SRSF3_1_F	CGTCGCCCTCGAGATGAT	NMD
	SRSF3_1_R	TGACTGGCTAATGCAGATTCAGA	
	SRSF3_3_F	CGTCGCCCTCGAGATGAT	Normal
	SRSF3_3_R	CGGCTGCGAGAGAAGCTT	
SRSF6	SRSF6_1_F	CGATCGCGACGGCTACA	NMD
	SRSF6_1_R	AAAGGCAGTCATCTTAGCCTCAGT	
	SRSF6_3_F	ATCGCGACGGCTACAGCTA	Normal
	SRSF6_3_R	CGTATTTGTCTCTGCCAGATGTTC	
HNRNPL	HNRNPL_For1	CTTGGGACTACACAAACCCCA	Normal
	HNRNPL_Rev1	GTGGGGCCCTCCATATTCTG	
	HNRNPL_For3	CTTGGGACTACACAAACCCCA	NMD
	HNRNPL_Rev3	TGGACGCTTCAACAGTGAGT	
RPS12	RBS12_For1	CTCATCCACGATGGCCTAGC	Normal
	RBS12_Rev1	CACAGTTGGATGCAAGCACA	
	RBS12_For2	AGGCATGGAGTTCATGGTGTT	NMD
	RBS12_Rev2	CACAGTTGGATGCAAGCACA	
HNRNPK	HNRNPK_For1	AGAATGCTGGGGCAGTGATT	Normal
	HNRNPK_Rev1	CACTGCTGTCTGGGACTGAA	
	HNRNPK_For2	AGAATGCTGGGGCAGTGATT	NMD
	HNRNPK_Rev2	TCCAAGGTAGGGATGATTTTCTTCA	
NEU1	NEU1_For1	GGTCAGCCCAAGCAGGAAAA	Normal
	NEU1_Rev1	GGTTTCGGGCATTGATGACG	
	NEU1_For2	GGTCAGCCCAAGCAGGAAAA	NMD
	NEU1-Rev2	CAGGGTCGAAGGTCACATCA	
SF1	SF1_For1	GCCTACAGTTATTCCCCCTGG	Normal
	SF1_Rev1	TGTGCGCAGTTTACGAGTCA	
	SF1_For2	GCCTACAGTTATTCCCCCTGG	NMD
	SF1_Rev2	AACTCTCGGGTGTTAAGCCG	
HPRT1	HPRT_For	GCTATAAATTCTTTGCTGACCTGCTG	for normalization
	HPRT_Rev	AATTACTTTTATGTCCCCTGTTGACTGG	
GAPDH	GAPDH Forward	GCATCTTCTTTTGCGTCGCC	
GAPDH	GAPDH Reverse	GACCAAATCCGTTGACTCCG	
Noxa	hNoxa_F1	GCTGGAAGTCGAGTGTGCTA	
Noxa	hNoxa_R1	GGAGTCCCCTCATGCAAGTT	
Puma	hPuma_total_F1	CCTGGAGGGTCCTGTACAATCT	
Puma	hPuma_total_R1	GCACCTAATTGGGCTCCATCT	
sXBP1	hXBP1_spliced_F	TGCTGAGTCCGCAGCAGG	
sXBP1	hXBP1_spliced_R	GTCCAGAATGCCCAACAGGA	
ATF4	hATF4_F	CCAGGTGTTCTCTGTGGGTC	
ATF4	hATF4_R	TGGCTGCTGTCTTGTTTTGC	
HSPA5	hHSPA5_F	TCACATGTCTTTGGGTGGGG	
HSPA5	hHSPA5_R	CCAGATGCACATGACCCAGT	
CHOP	hCHOP_forward	GGAGCATCAGTCCCCCACTT	
CHOP	hCHOP-reverse	TGTGGGATTGAGGGTCACATC	
ATF3	hATF3_forward	CTGCAGAAAGAGTCGGAG	
ATF3	hATF3_reverse	TGAGCCCGGACAATACAC	

Table S4: GI50 and Emax for 141 cell lines in a 3 day proliferation assay treated with CC-115 and CC-223

Group 1						
		CC-115		CC-223		
Cell Line	RRID	GI50	Emax	GI50	Emax	delta Emax
OCI-Ly7	CVCL_1881	176.0	-100.0	632.7	-80.3	-19.7
Malme-3	CVCL_1437	116.3	-52.7	105.7	-33.3	-19.3
NCI-H727	CVCL_1584	241.6	-5.8	275.8	13.5	-19.3
HCC1500	CVCL_1254	5.0	-75.0	27.0	-56.0	-19.0
Karpas-422	CVCL_1325	180.5	-7.5	549.5	10.9	-18.4
CAL-120	CVCL_1104	182.0	-8.2	238.7	10.0	-18.2
NCI-H2030	CVCL_1517	105.3	-36.3	105.0	-18.3	-18.0
Huh-7	CVCL_0336	47.5	-38.5	80.5	-20.5	-18.0
MDA-MB-436	CVCL_0623	247.2	17.0	474.8	34.2	-17.2
CCD-1112Sk	CVCL_2769	151.5	-5.0	324.5	11.5	-16.5
Karpas-231	CVCL_1822	144.7	-100.0	915.3	-84.0	-16.0
FaDu	CVCL_1218	792.7	15.3	1573.0	30.3	-15.0
Pfeiffer	CVCL_3326	86.5	-97.3	78.3	-83.5	-13.8
SK-HEP-1	CVCL_0525	113.3	-16.7	267.7	-3.3	-13.3
DU4475	CVCL_1183	237.3	-100.0	1103.5	-86.7	-13.3
NCI-H647	CVCL_1574	180.0	-44.0	217.3	-31.3	-12.7
MC116	CVCL_1399	267.5	-100.0	1233.0	-88.0	-12.0
THP-1	CVCL_0006	226.7	-10.7	215.3	1.3	-12.0
NCI-H838	CVCL_1594	181.5	11.0	477.8	22.8	-11.8
NCI-H520	CVCL_1566	154.0	-2.5	174.5	8.5	-11.0
WSU-DLCL2	CVCL_1902	459.5	-99.5	1084.5	-89.0	-10.5
WSU-FSCCL	CVCL_1903	156.0	-100.0	215.7	-89.7	-10.3
A-253	CVCL_1060	303.0	16.0	320.0	26.0	-10.0
CCD-18Co	CVCL_2379	112.3	-37.7	198.0	-27.7	-10.0
NCI-H1395	CVCL_1467	57.3	0.3	62.2	10.0	-9.8
Hs 578T	CVCL_0332	285.7	2.3	422.3	11.3	-9.0
U-2940	CVCL_1897	142.2	-52.5	149.2	-44.8	-7.7
Mino	CVCL_1872	70.5	-99.5	560.5	-92.5	-7.0
KG-1	CVCL_0374	506.3	-6.0	588.0	0.7	-6.7
CAL-148	CVCL_1106	187.3	-100.0	348.3	-95.7	-4.3
Kasumi-1	CVCL_0589	39.3	-100.0	63.7	-99.3	-0.7
SU-DHL-10	CVCL_1889	229.4	-99.6	852.3	-99.3	-0.3
RPMI-2650	CVCL_1664	218.3	1.3	346.3	1.7	-0.3
DoHH2	CVCL_1179	133.7	-100.0	237.7	-99.7	-0.3
WSU-NHL	CVCL_1793	47.3	-100.0	73.7	-99.7	-0.3
SU-DHL-6	CVCL_2206	56.2	-99.8	149.4	-99.6	-0.2
SU-DHL-16	CVCL_1890	83.0	-100.0	248.5	-100.0	0.0
SU-DHL-5	CVCL_1735	168.0	-100.0	419.0	-100.0	0.0
BT-474	CVCL_0179	75.0	-12.0	219.5	-12.5	0.5

Group 2						
		CC-115		CC-223		
Cell Line	RRID	GI50	Emax	GI50	Emax	delta Emax
HCT 116	CVCL_0291	422.5	-79.0	469.0	38.5	-117.5
HCC70	CVCL_1270	141.5	-89.0	166.5	26.0	-115.0
SU-DHL-4	CVCL_0539	322.7	-88.6	562.7	5.9	-94.5
SNU-449	CVCL_0454	188.3	-91.7	368.3	1.7	-93.3
Hep 3B2.1-7	CVCL_0326	135.7	-97.0	255.0	-5.7	-91.3
HCC38	CVCL_1267	429.8	-90.0	987.0	-1.7	-88.3
OCI-Ly10	CVCL_8795	176.6	-80.8	379.6	7.4	-88.2
HCC1143	CVCL_1245	399.7	-76.0	471.0	11.6	-87.6
CAL-33	CVCL_1108	43.0	-93.3	38.0	-8.3	-85.0
MDA-MB-157	CVCL_0618	437.0	-70.0	669.7	14.0	-84.0
Daudi	CVCL_0008	134.7	-86.0	204.0	-3.4	-82.7
CAL-51	CVCL_1110	70.7	-92.3	113.0	-10.2	-82.1
SU-DHL-1	CVCL_0538	323.7	-57.0	1357.0	24.0	-81.0
HCC1806	CVCL_1258	264.8	-69.3	293.7	11.7	-80.9
U-2932	CVCL_1896	277.5	-70.5	881.8	10.4	-80.9
NCI-H1650	CVCL_1483	714.0	-42.5	2285.5	33.5	-76.0
NCI-H1792	CVCL_1495	158.7	-67.3	217.3	7.3	-74.6
MCF-7	CVCL_0031	68.0	-67.0	180.5	7.5	-74.5
CAL-27	CVCL_1107	144.3	-50.3	150.0	23.7	-74.0
SNU-398	CVCL_0077	67.0	-67.0	283.7	6.0	-73.0
HT	CVCL_1290	214.9	-67.1	390.3	5.2	-72.3
Ramos	CVCL_0597	260.0	-100.0	429.5	-29.5	-70.5
HDQ-P1	CVCL_2067	162.0	-51.7	235.0	18.7	-70.3
NCI-H1975	CVCL_1511	205.0	-62.0	253.0	8.0	-70.0
OCI-Ly19	CVCL_1878	301.8	-64.3	758.8	5.3	-69.5
SCC-9	CVCL_1685	129.0	-40.3	590.3	28.3	-68.7
NCI-H1838	CVCL_1499	438.5	-44.0	1229.0	23.0	-67.0
SU-DHL-8	CVCL_2207	128.0	-91.0	250.0	-25.0	-66.0
DB	CVCL_1168	246.3	-60.2	316.4	5.7	-65.9
HOP-92	CVCL_1286	193.8	-53.5	265.0	11.4	-64.9
Farage	CVCL_3302	325.0	-97.7	593.3	-32.9	-64.8
HCC1937	CVCL_0290	212.5	-50.0	74.0	14.5	-64.5
MT-3	CVCL_2129	149.7	-81.3	367.3	-17.0	-64.3
NCI-H23	CVCL_1547	305.3	-43.3	461.0	19.7	-63.0
BT-20	CVCL_0178	175.0	-86.0	247.0	-23.8	-62.2
PLC/PRF/5	CVCL_0485	143.7	-59.0	309.3	2.7	-61.7
NCI-H441	CVCL_1561	530.0	-23.8	432.0	36.7	-60.4
Hep-G2	CVCL_0027	141.0	-29.0	244.7	30.0	-59.0
A-549	CVCL_0023	210.0	-51.5	296.0	7.5	-59.0
NCI-H460	CVCL_0459	86.6	-50.0	122.2	8.0	-58.0
NCI-H596	CVCL_1571	122.3	-83.7	110.7	-26.7	-57.0
HOP-62	CVCL_1285	341.0	-66.0	414.0	-10.0	-56.0
NCI-H28	CVCL_1555	81.5	-55.0	72.5	0.5	-55.5
NCI-H2110	CVCL_1530	147.3	-68.7	203.0	-13.3	-55.3
NCI-H2122	CVCL_1531	66.7	-77.3	70.0	-23.0	-54.3
JVM-13	CVCL_1318	1771.7	-7.3	1946.7	46.7	-54.0
SNU-182	CVCL_0090	36.7	-93.7	83.0	-42.3	-51.3
TMD8	CVCL_A442	161.3	-99.7	190.0	-48.7	-51.0
Calu-1	CVCL_0608	163.7	-58.0	142.3	-7.0	-51.0
NCI-H1563	CVCL_1475	124.0	-55.5	191.7	-5.3	-50.2
JeKo-1	CVCL_1865	35.0	-98.0	76.0	-48.7	-49.3
EKVX	CVCL_1195	475.3	-51.0	512.7	-1.7	-49.3
SNU-423	CVCL_0366	162.3	-77.3	299.7	-29.3	-48.0
NCI-H1299	CVCL_0060	182.7	-38.0	221.3	10.0	-48.0
CAL-85-1	CVCL_1114	89.0	-51.3	163.0	-3.5	-47.8
NCI-H1993	CVCL_1512	85.5	-68.0	85.0	-21.0	-47.0
SCC-25	CVCL_1682	127.3	-35.0	174.3	10.3	-45.3
NCI-H1755	CVCL_1492	88.3	-86.0	103.7	-40.7	-45.3
NCI-H1437	CVCL_1472	282.7	-57.0	418.7	-11.7	-45.3
HL-60	CVCL_0002	237.0	-39.3	459.7	5.0	-44.3
MRC-5	CVCL_0440	130.3	-39.3	195.0	5.0	-44.3
MDA-MB-231	CVCL_0062	194.0	-30.3	546.0	13.5	-43.8
Namalwa	CVCL_0067	126.0	-96.3	645.3	-54.0	-42.3
HCC1428	CVCL_1252	233.0	-45.0	98.5	-3.0	-42.0
SNU-387	CVCL_0250	309.0	-27.7	1524.3	13.7	-41.3
Calu-3	CVCL_0609	148.3	-57.7	152.4	-17.2	-40.5
Karpas-299	CVCL_1324	309.0	-46.0	522.3	-6.3	-39.7
SCC-15	CVCL_1681	56.7	-49.0	32.0	-9.5	-39.5
HCC2157	CVCL_1261	51.7	-100.0	51.6	-60.8	-39.2
MDA-MB-453	CVCL_0418	386.0	-39.0	138.5	0.0	-39.0
UACC-812	CVCL_1781	259.0	-75.0	253.0	-36.4	-38.6
Detroit 562	CVCL_1171	169.0	-40.7	95.7	-2.3	-38.3
RC-K8	CVCL_1883	214.0	-15.5	343.5	22.5	-38.0
NCI-H1703	CVCL_1490	156.7	-49.0	108.3	-11.3	-37.7
SK-BR-3	CVCL_0033	111.3	-37.0	59.3	0.0	-37.0
MDA-MB-157	CVCL_0618	345.8	-98.5	224.7	-61.7	-36.8
NCI-H2444	CVCL_1552	234.0	-91.0	995.7	-54.7	-36.3
JVM-2	CVCL_1319	247.3	-13.8	306.3	22.0	-35.8
OCI-Ly3	CVCL_8800	154.0	-88.0	161.5	-52.5	-35.5
NCI-H2228	CVCL_1543	164.0	-26.0	255.7	9.0	-35.0
Toledo	CVCL_3611	271.5	-30.5	238.5	2.5	-33.0
MOLM-13	CVCL_2119	105.0	-93.7	137.7	-61.0	-32.7
NCI-H358	CVCL_1559	416.7	-31.3	683.0	0.0	-31.3
NCI-H2009	CVCL_1514	155.0	-38.0	140.5	-7.5	-30.5
SC-1	CVCL_3435	299.3	-2.7	758.7	27.5	-30.2
SK-LU-1	CVCL_0629	57.5	-27.0	76.0	2.5	-29.5
REC-1	CVCL_1884	161.6	-100.0	1058.4	-70.6	-29.4
NCI-H226	CVCL_1544	134.8	-35.0	186.6	-5.8	-29.2
NCI-H1944	CVCL_1508	90.5	-45.0	124.0	-16.0	-29.0
BHY	CVCL_1086	39.5	-69.0	78.5	-40.5	-28.5
MDA-MB-468	CVCL_0419	131.0	-72.7	465.7	-44.3	-28.3
NCI-H1568	CVCL_1476	96.0	-46.3	89.0	-18.3	-28.0
BT-549	CVCL_1092	215.8	-76.8	182.0	-49.0	-27.8
SNU-475	CVCL_0497	156.0	-50.7	464.7	-24.7	-26.0
HN	CVCL_1283	117.0	-30.0	230.0	-4.3	-25.7
Karpas-1106P	CVCL_1821	198.0	-99.2	456.3	-74.7	-24.5
Ri-1	CVCL_1885	115.5	-97.5	333.0	-75.5	-22.0
Calu-6	CVCL_0236	245.0	-27.7	230.7	-5.7	-22.0
SW1573	CVCL_1720	252.5	-0.5	374.0	21.5	-22.0
Granta-519	CVCL_1818	337.6	2.2	447.8	24.0	-21.8
T-47D	CVCL_0553	51.0	-13.0	80.0	7.8	-20.8
NU-DHL-1	CVCL_1876	154.7	-100.0	150.7	-80.0	-20.0

Group 2A

Group 2B

delta Emax = CC-115 Emax - CC-223 Emax

Figure S1: SMG1i does not inhibit phosphorylation of DNA-PK

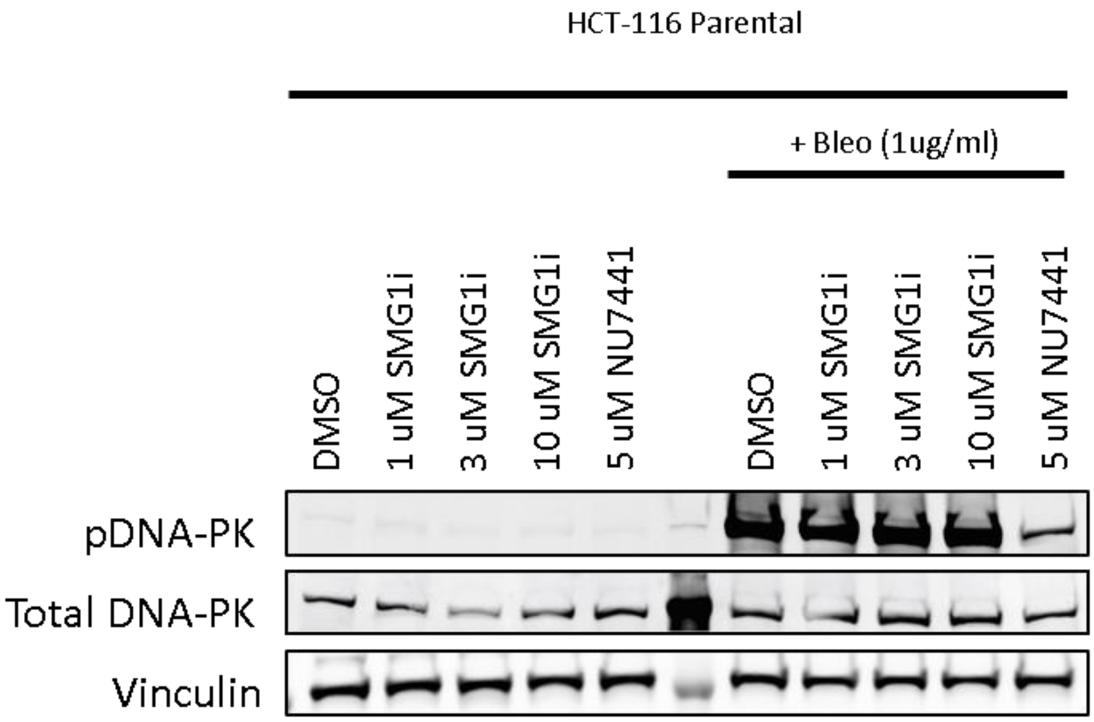

Figure S2: Block titration of CC-115 plus ABT-199 in three CLL cell lines

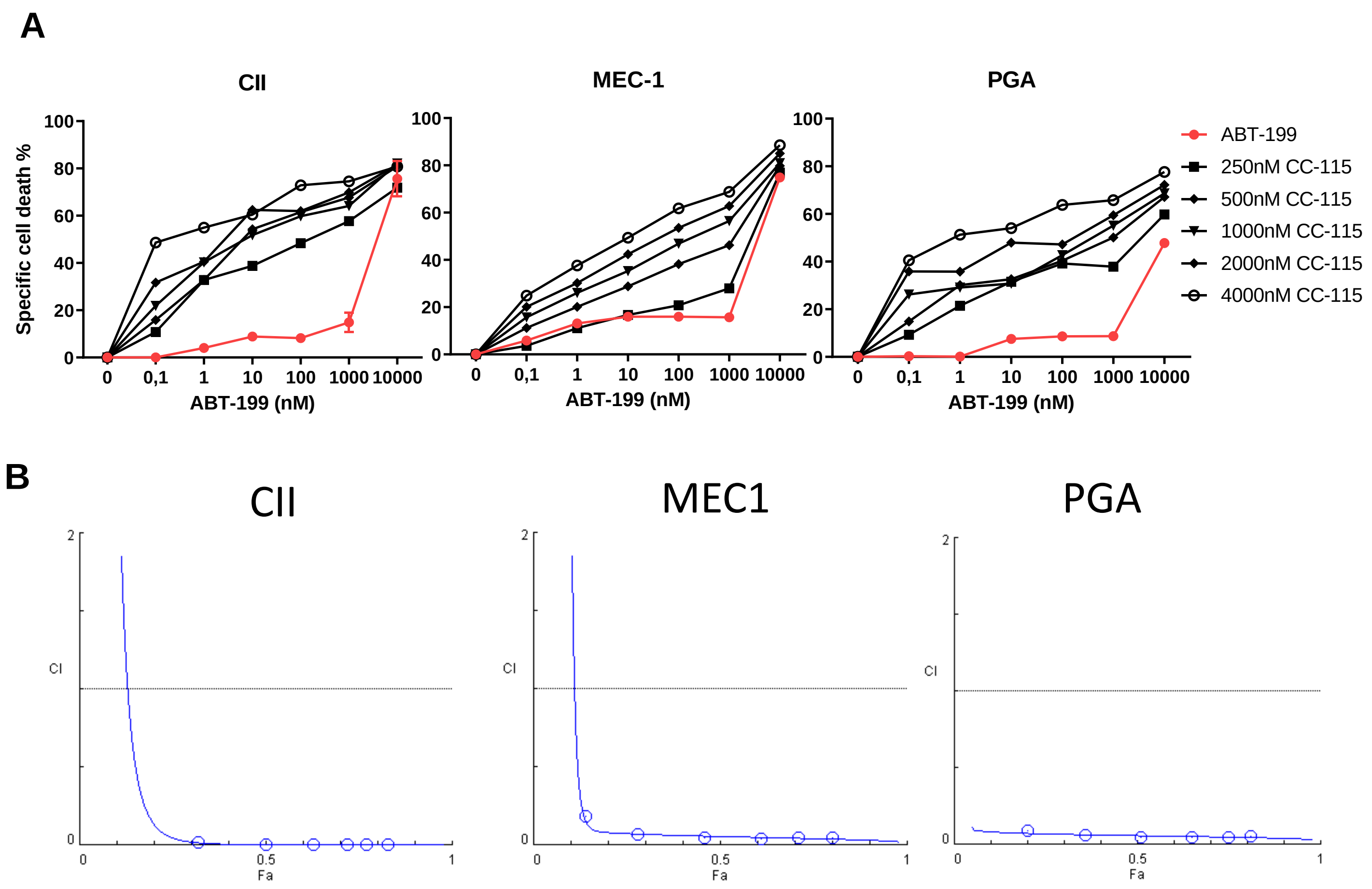

Figure S3: Effect of CC-115 on viability in comparison to DNA-PK and/or TORK inhibition in MM cell lines.

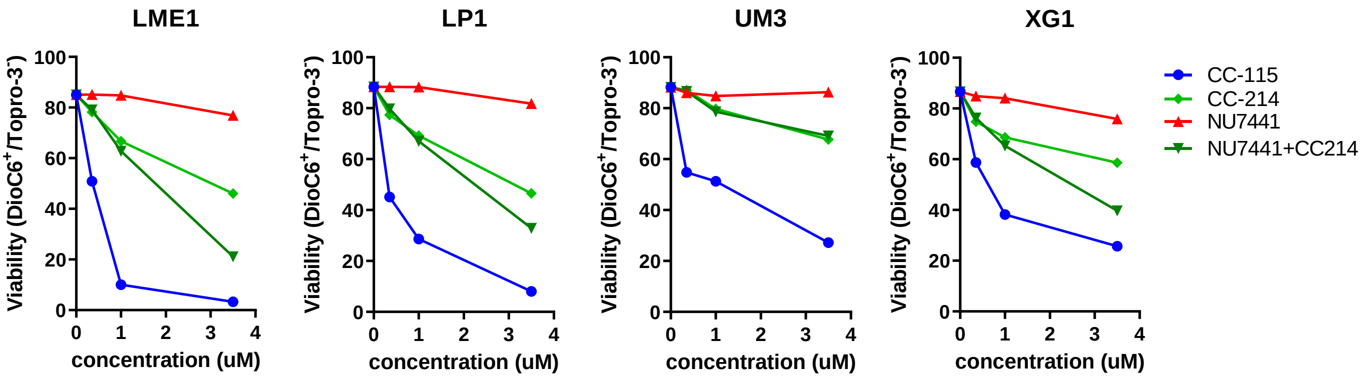

Figure S4: Effect CC-115 on UPR related transcripts

A

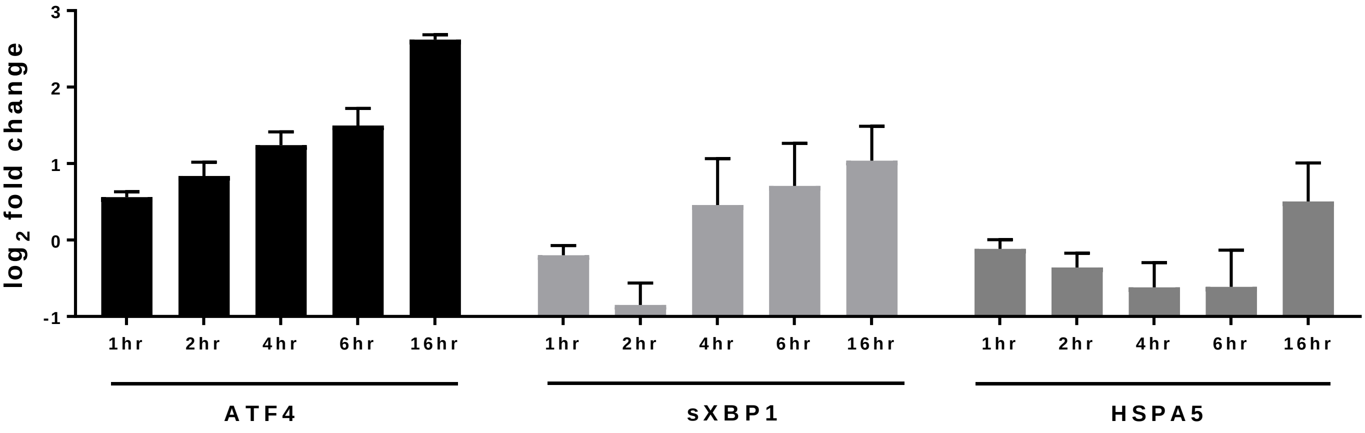

B

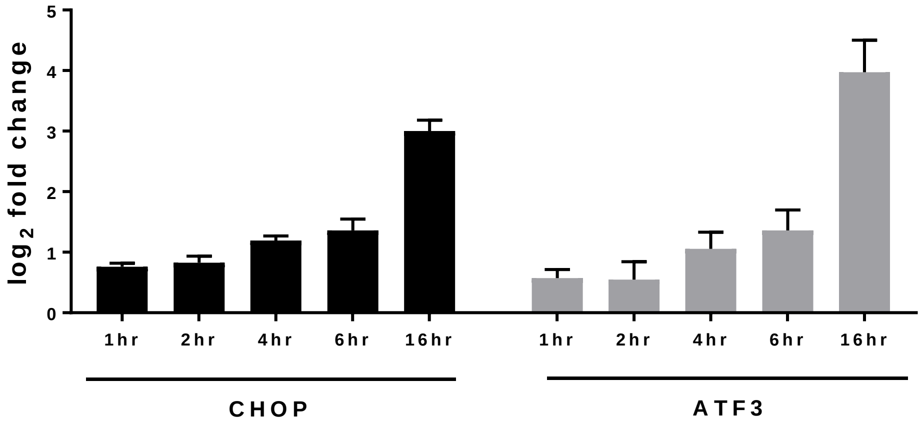

C

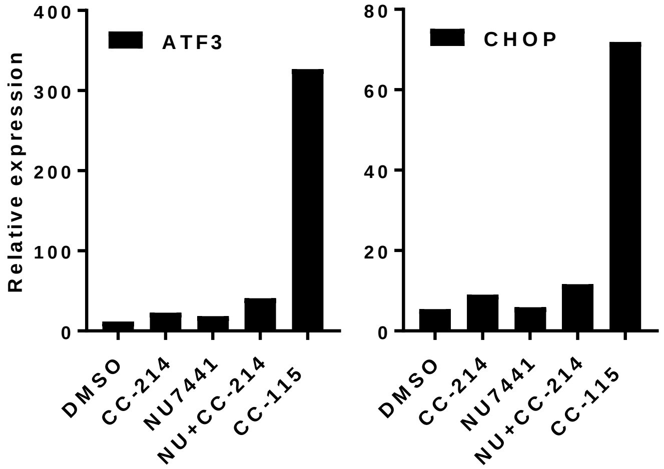

Figure S5: Validation of different CRISPR knock-out cell lines by western blot

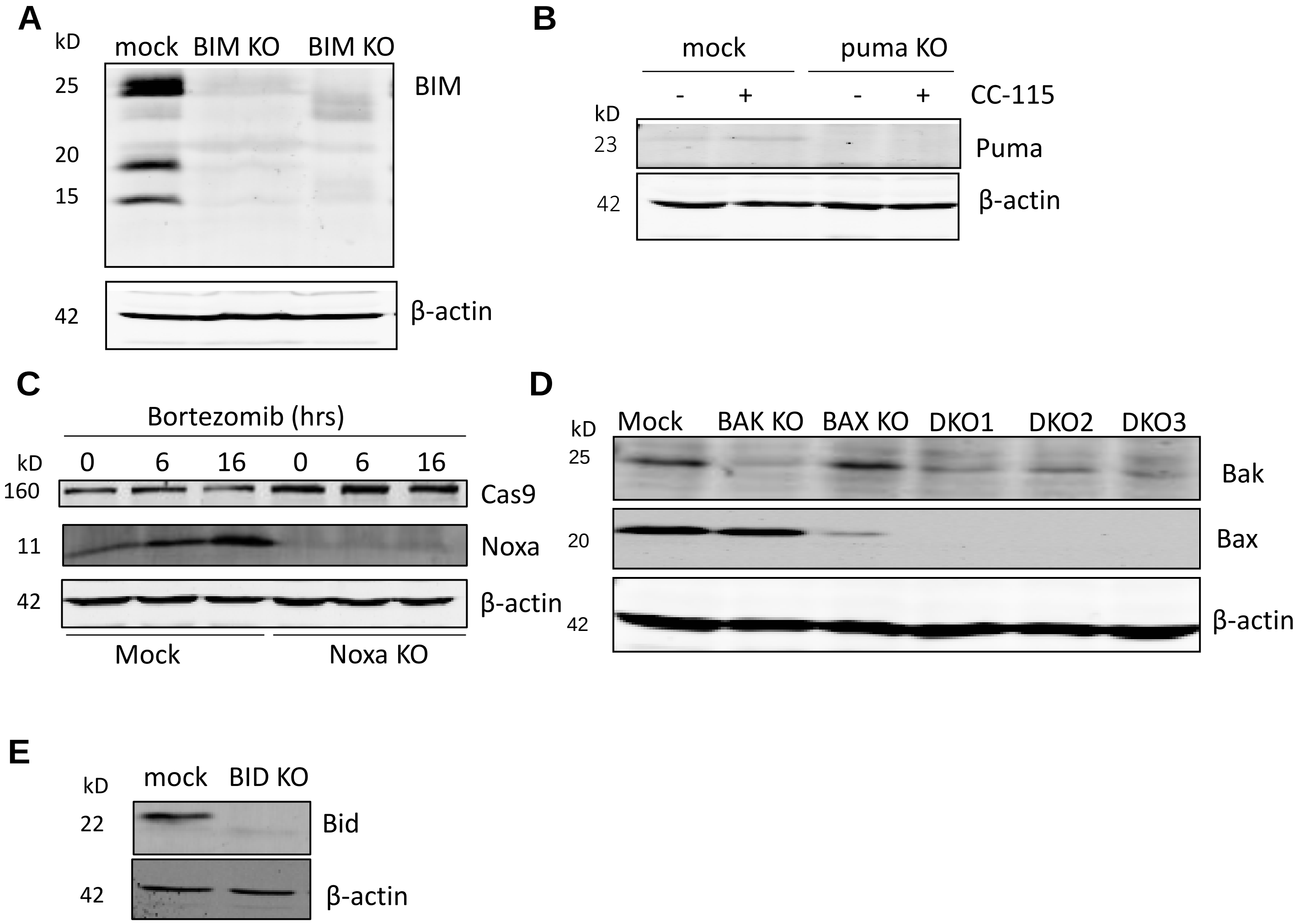

Figure S6: CC-115 inhibits SMG1 *in vivo*

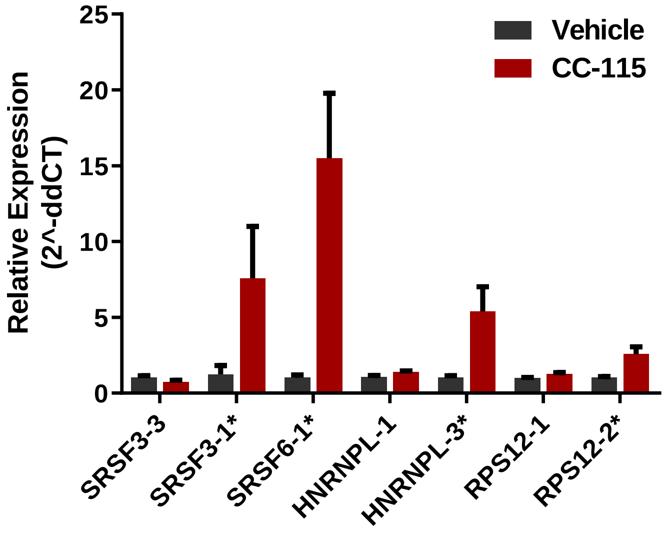

Figure S7: Bodyweight monitoring in *in vivo* mouse experiments - related to Figure 5

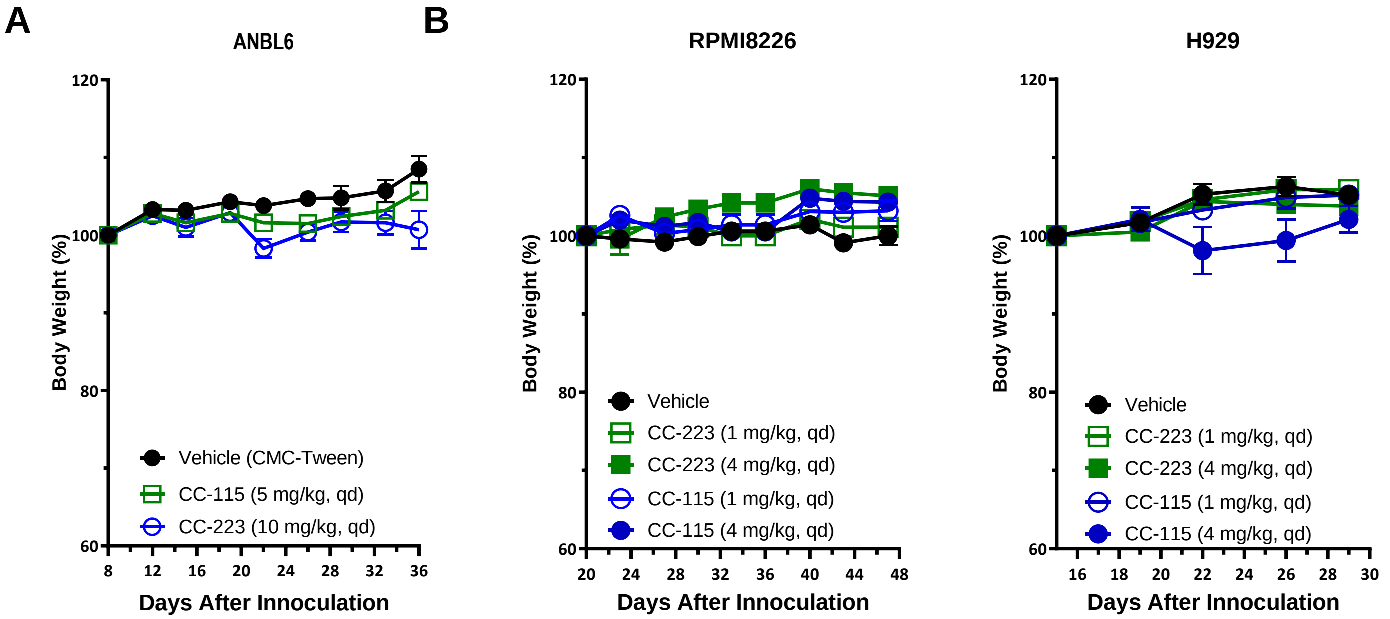

Supplement: Supplementary file 1 — Table S1. Single guide RNAs used for generation of different CRISPR/Cas9 knockout cells. Table S2. Antibodies used for western blotting analysis. Table S3. Primers used for Real‐Time quantitative PCR. Table S4. GI50 and Emax for 141 cell lines in a 3‐day proliferation assay treated with CC‐115 and CC‐223. Table S5. Results from ActivX KiNativ analysis comparing different doses of CC‐115 or CC‐223 in four different Group 2B cell lines. Fig. S1. SMG1i does not inhibit the Ser2056 phosphorylation of DNA‐PK. Fig. S2. Block titration of CC‐115 plus ABT‐199 in three CLL cell lines. Fig. S3. Effect of CC‐115 on viability in comparison with DNA‐PK and/or TORK inhibition in MM cell lines. Fig. S4. Effect of CC‐115 on UPR‐related transcripts. Fig. S5. Validation of different KO cell lines by western blotting. Fig. S6. qPCR analysis on HCT 116 xenograft tumors treated with Vehicle or CC‐115 (qPCR normalized to control gene HPRT1 and relative to vehicle). Fig. S7. Bodyweight monitoring in in vivo mouse experiments, related to Fig. 5. [file MOL2-17-284-s001.zip › mol213343-sup-0001-Supinfo.pdf]
