# Supplementary material for: SMG1, a nonsense‐mediated mRNA decay (NMD) regulator, as a candidate therapeutic target in multiple myeloma
Source: Mol Oncol. 2022 Dec 16;17(2):284–97. doi: 10.1002/1878-0261.13343 (PMC9892823; doi:10.1002/1878-0261.13343)
Supplement: Supplementary file 1 — Table S1. Single guide RNAs used for generation of different CRISPR/Cas9 knockout cells. Table S2. Antibodies used for western blotting analysis. Table S3. Primers used for Real‐Time quantitative PCR. Table S4. GI50 and Emax for 141 cell lines in a 3‐day proliferation assay treated with CC‐115 and CC‐223. Table S5. Results from ActivX KiNativ analysis comparing different doses of CC‐115 or CC‐223 in four different Group 2B cell lines. Fig. S1. SMG1i does not inhibit the Ser2056 phosphorylation of DNA‐PK. Fig. S2. Block titration of CC‐115 plus ABT‐199 in three CLL cell lines. Fig. S3. Effect of CC‐115 on viability in comparison with DNA‐PK and/or TORK inhibition in MM cell lines. Fig. S4. Effect of CC‐115 on UPR‐related transcripts. Fig. S5. Validation of different KO cell lines by western blotting. Fig. S6. qPCR analysis on HCT 116 xenograft tumors treated with Vehicle or CC‐115 (qPCR normalized to control gene HPRT1 and relative to vehicle). Fig. S7. Bodyweight monitoring in in vivo mouse experiments, related to Fig. 5. [file MOL2-17-284-s001.zip › mol213343-sup-0002-Legends.docx]

**Supporting Information**

**Supplemental Table 1.** Single guide RNAs used for generation of different CRISPR/Cas9 knock-out cells.

**Supplemental Table 2.** Antibodies used for western blotting analysis.

**Supplemental Table 3.** Primers used for Real Time quantitative PCR.

**Supplemental Table 4**. GI50 and Emax for 141 cell lines in a 3 day proliferation assay treated with CC-115 and CC-223. Cell lines were grouped based on cellular response to TORK inhibitors CC-223 and CC-115 based on delta Emax (group 1 delta Emax>-20 and group 2 delta Emax<-20). Emax was defined as maximum inhibition of proliferation after 72 hours incubation as described in methods. A negative delta Emax indicates a higher activity of CC-115 compared to CC-223.

**Supplemental Table 5**. Results from ActivX KiNativ analysis comparing different doses of CC-115 or CC-223 in four different Group 2B cell lines.

**Supplemental Figure 1**. SMG1i does not inhibit the Ser2056 phosphorylation of DNA-PK. Western blot analysis of HCT 116 parental cell line with and without 1 µg/ml bleomycin (bleo) stimulation treated with DMSO, SMG1i, and NU7441 at indicated doses.

**Supplemental Figure 2.** Block titration of CC-115 plus ABT-199 in three CLL cell lines. A. Cells were treated for 48 hours with various concentrations either as single drug ABT-199 (red) or a combination of CC-115 with ABT-199 (black) and stained with DIOC6/TOPRO-3. Specific cell death was defined as ([% cell death in treated cells] – [% cell death in medium control]) / [% viable cells medium control]) x 100]). B. Fraction affected-Combination index (Fa-CI) plots of CLL cell lines treated with a combination of CC-115 and venetoclax (ABT-199) showing the Chou-Talalay combination index (CI) as a function of the fraction of affected (Fa) after a combination treatment with CC-115 and ABT-199 at various concentrations. Horizontal lines represent CI = 1, signifying additive effects. Datapoints represent CI values at different drug concentrations.

**Supplemental Figure 3**. Effect of CC-115 on viability in comparison to DNA-PK and/or TORK inhibition in MM cell lines. MM cell lines (LME1, LP1, UM3 and XG1 ) in which CC-115 had more activity than the combination of a TORK inhibitor (CC214-1) with a DNA-PK inhibitor (NU7441). Cells were treated for 48 hours and stained with DIOC6/TOPRO-3.

**Supplemental Figure 4**. Effect CC-115 on UPR related transcripts. A. Effect of CC-115 treatment (1µM in the presence of QVD 5µM) on *ATF4*, *sXBP1* and *HSPA5* transcription for indicated time points. Shown are the results of qPCR analysis. Relative expression was calculated by the comparative ΔCt method. Shown are bar plots with fold induction (treated vs. untreated) on the y-axis. Expression was normalized to *GAPDH*. Error bars represent ± SEM of three different MM cell lines (LME1, LP1 and RPMI8226). B. Similar to (A) for *ATF3* and *CHOP* transcription. C. Effect of CC-115 on *ATF3* and *CHOP* mRNA expression is independent of TORK and/or DNA-PK inhibition. RPMI8226 cells were incubated in the presence of TORK inhibition by CC-214 (1µM), DNA-PK inhibition by NU7441 (1µM), the combination NU7441 (1µM)+ CC-214 (1µM), CC-115 (1µM) or equal amounts of DMSO.

**Supplemental Figure 5**. Validation different KO cell lines by western blotting. Western blot analyses to confirm different RPMI8226 CRISPR KO cell lines used for experiments shown in Figure 4. A. Confirmation BIM KO. B. Effect of CC-115 (16 hours 1µM) on puma levels in mock and PUMA KO cells. C. Effect of bortezomib (20nM) treatment on NOXA protein levels in NOXA KO cells. D. Confirmation BAK, BAX and BAX/BAK double KO (DKO) on Bax and Bak analyzed by Western blotting. E. Confirmation BID KO.

**Supplemental Figure 6**. qPCR analysis on HCT 116 xenograft tumors treated with Vehicle or CC-115 (qPCR normalized to control gene HPRT1 and relative to Vehicle). Bars represent average for 4 tumors and error bars represent SD. * = NMD Transcript.

**Supplemental Figure 7.** Bodyweight monitoring in *in vivo* mouse experiments - related to Figure 5. Body weights of mice measured at different time points after tumor cell inoculation with normalized body weight in percentage on the y-axis and days after tumor cell inoculation on the x-axis.
